# Effectiveness of 24-h mobile reporting tool during a malaria outbreak in Mpumalanga Province, South Africa

**DOI:** 10.1186/s12936-019-2683-4

**Published:** 2019-02-21

**Authors:** Craig Davies, Rebecca Graffy, Mbavhalelo Shandukani, Ednah Baloyi, Laura Gast, Gerdalize Kok, Frans Mbokazi, Alpheus Zita, Mandla Zwane, Ray Magagula, Aaron Mabuza, Wayne Ramkrishna, Natashia Morris, Jacqueline Porteous, George Shirreff, Lucille Blumberg, Eunice Misiani, Devanand Moonasar

**Affiliations:** 1 Malaria Programme, Clinton Health Access Initiative, Pretoria, South Africa; 2 Department of Health, Malaria and Other Vector Borne Diseases, Pretoria, South Africa; 3 Mpumalanga Department of Health, Malaria Control Programme, Mbombela, South Africa; 4 Centre for Disease Control, Mpumalanga Department of Health and Social Development, Mbombela, South Africa; 5 Medical Research Council, Durban, South Africa; 6 Division of Public Health Surveillance and Response, National Institute for Communicable Diseases (NICD) of the National Health Laboratory Service (NHLS), Johannesburg, South Africa; 7 School of Health Systems and Public Health, University of Pretoria, Pretoria, South Africa

**Keywords:** Malaria, South Africa, Surveillance, Outbreak, Mobile reporting, MalariaConnect

## Abstract

**Background:**

As surveillance is a key strategy for malaria elimination in South Africa, ensuring strong surveillance systems is a National Department of Health priority. Historically, real time tracking of case trends and reporting within 24 h—a requirement in South Africa’s National surveillance guidelines—has not been possible. To enhance surveillance and response efficiency, a mobile surveillance tool, MalariaConnect, was developed using Unstructured Supplementary Service Data (USSD) technology. It was rolled out in health facilities in malaria endemic areas of South Africa to provide 24-h reporting of malaria cases.

**Methods:**

To evaluate the efficiency of the mobile tool to detect an outbreak data were extracted from the paper based and MalariaConnect reporting systems in Bushbuckridge from 1 January to 18 June 2017. These data were subject to time series analyses to determine if MalariaConnect provided sufficient data reliably to detect increasing case trends reported through the paper system. The Chi squared test was used to determine goodness of fit between the following indicator data generated using MalariaConnect and paper reporting systems: timeliness, completeness, and precision.

**Results:**

MalariaConnect adequately tracked case trends reported through the paper system. Timeliness of reporting increased significantly using MalariaConnect with 0.63 days to notification compared to 5.65 days using the paper-system (p < 0.05). The completeness of reporting was significantly higher for the paper system (100% completion; p < 0.05), compared to confirmed MalariaConnect cases (61%). There was a moderate association between data precision and the reporting system (p < 0.05). MalariaConnect provided an effective way of reliably and accurately identifying the onset of the malaria outbreak in Bushbuckridge.

**Conclusion:**

Timeliness significantly improved using MalariaConnect and in a malaria elimination setting, can be used to markedly improve case investigation and response activities within the recommended 72-h period. Although data completeness and precision were lower compared to paper reporting, MalariaConnect data can be used to trigger outbreak responses.

## Background

The north-eastern region of South Africa is at a continuous risk of malaria epidemics attributable to unstable transmission dynamics and low immunity among the population [1]. The World Health Organization (WHO) [2] estimates that out of the total population of 54.5 million in South Africa, approximately 2.18 million individuals (4%) live in high transmission areas (> 1 case per 1000 population) and 3.27 million (6%) live in low transmission areas (0–1 cases per 1000). Malaria has been listed as a priority disease for the NDoH in South Africa [3], which, through robust implementation of key interventions, has resulted in significant reductions in the burden of malaria in the past two decades. In the year 2001, 25,731 malaria cases were reported in the three endemic provinces of KwaZulu-Natal, Limpopo and Mpumalanga. By 2016, the number of reported malaria cases had declined by 83%, to 4252 cases [4]. This decline has been driven by successful indoor residual spraying campaigns in the endemic provinces, the free provision of artemisinin-based combination therapy (ACT) in public health facilities, and the added effect of strengthening regional malaria control programmes. Given these achievements in reducing malaria incidence, South Africa has moved from “control” towards “elimination” status, as defined by the WHO [5] criteria, and is one of 21 endemic countries that have the potential to eliminate malaria within their borders by 2020 [2].

A key pillar of South Africa’s strategy to eliminate malaria is the strengthening of surveillance systems to ensure routine and prompt reporting of malaria cases [1]. The collection of good quality, timely, high resolution disease surveillance data supports decision-making through rapid generation of real-time information, and will enable more effective public health response [6]. This drive toward efficient reporting systems is critical not only for reduction of malaria burden, but also for verification of elimination status and prevention of reintroduction of transmission.

An ideal surveillance system will support the collection of real-time, accurate case incidence for routine tracking, case classification, and will facilitate early identification of potential outbreaks through case trends. This ideal surveillance system must be supported through robust data collection that: (1) matches the intended purpose of monitoring case trends, individually and in relation to epidemic preparedness and response criteria (data quality and validity), (2) are collected and reported in the appropriate timeframes (timeliness), (3) are complete and with no missing reports (completeness), and (4) are free of random error (precision) [5].

Within the elimination setting, timeliness of reporting becomes increasingly critical, and reporting of cases should approach real-time reporting of individual geolocated cases to halt continuing transmission or reintroduction into a formerly cleared area [7]. In South Africa, a paper-based case reporting system has traditionally been used as the primary means for data collection for malaria surveillance. However, prompt tracking of malaria case trends has been a challenge due to delays both in data collection (completion of paper report forms) as well as in data recording (delivery of paper forms to district offices and recording of that data into the digital surveillance system). Consequently, South Africa has not been achieving case surveillance monitoring and evaluation timeliness targets as outlined by the WHO for countries in a malaria elimination setting—namely, notification within 24 h, verification within 48 h, and investigation and response beginning within 72 h [5].

A cellular network based, rapid-reporting system was created to address the issue of surveillance system timeliness within South Africa. The tool, called MalariaConnect, was developed with the explicit aim of facilitating rapid reporting of cases, and to facilitate rapid case investigation, case classification, and appropriate response planning. MalariaConnect is a USSD-based system and allows anyone to send in a report using any mobile phone at no cost to the user. By using the cellular network, the MalariaConnect system is designed to be fast, convenient, and user-friendly.

The MalariaConnect reporting system was rolled out in October 2015 in health facilities in high malaria burden districts across the three endemic provinces of South Africa: KwaZulu-Natal, Limpopo and Mpumalanga.

**Fig. 1:**
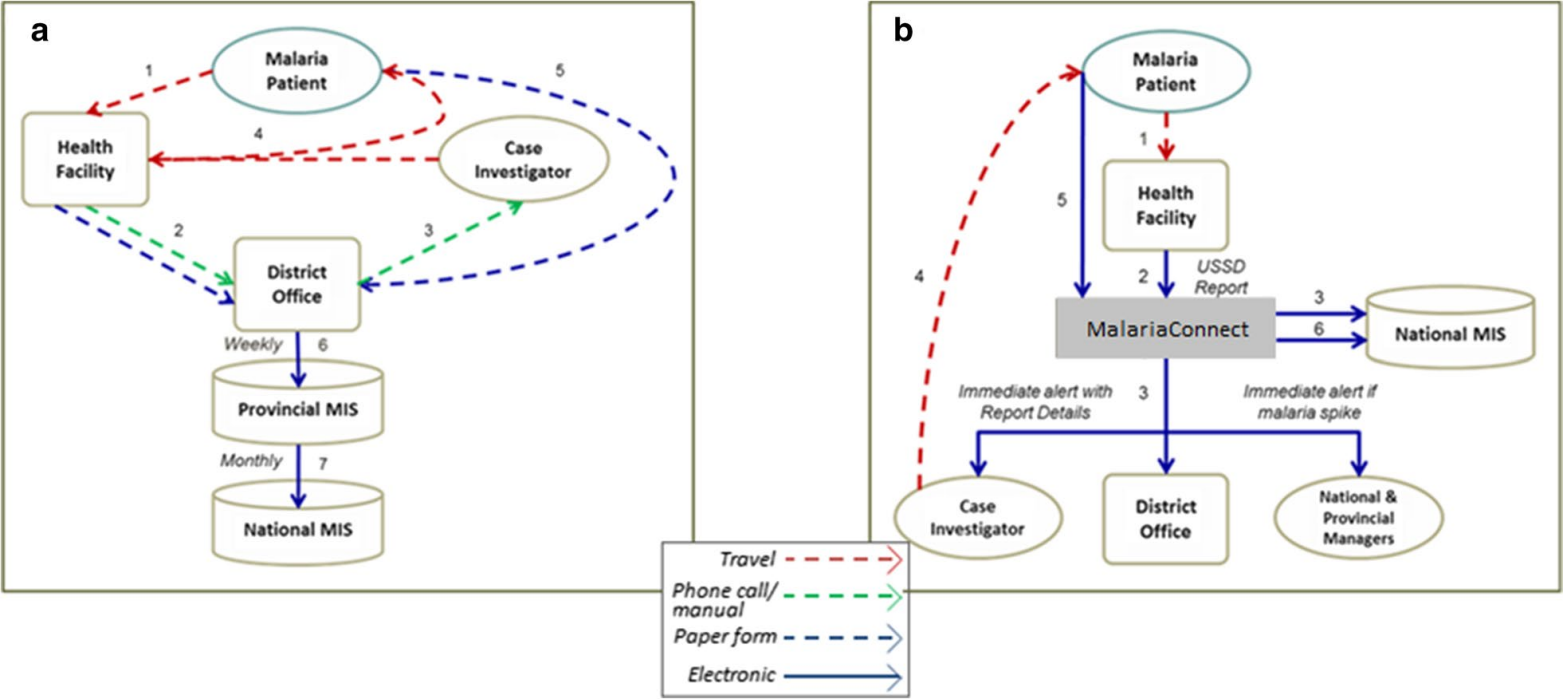
Flow of information when a malaria patient presents at a health facility and a malaria case notification is created for **a** paper-based reporting system and **b** MalariaConnect reporting system

Reporting of a malaria case is as follows: when a malaria case is detected at a facility, the case notification paper form is completed at all facilities, and the MalariaConnect notification is completed in those facilities that have received training on use of this tool. Paper notifications are collected from health facilities by case investigators and transported to the district data capturing office where they are entered directly into the Malaria Information System (MIS). MalariaConnect case notifications are captured directly into the web-based database and are also sent directly by email and Short Message Service (SMS) to the case investigator, environmental health practitioner, information officers, and programme managers responsible for the source health facility of the case (Fig. 1). Cases reported through MalariaConnect undergo validation to match these with the cases reported by paper.

**Fig. 2:**
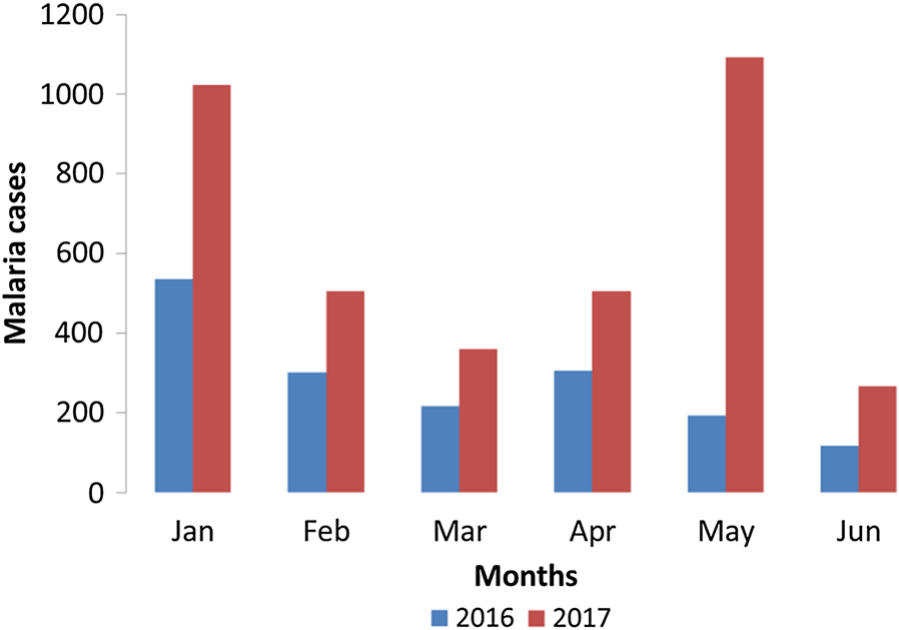
Comparison of malaria cases reported in Bushbuckridge, Mpumalanga through the paper reporting system over the first 6 months of 2016 versus 2017

An opportunity to test the operational performance of the MalariaConnect system versus the standard paper-based reporting system occurred in early 2017. The declaration of an outbreak in Bushbuckridge sub-district on 10th May 2017 was precipitated by a substantial increase in the trend of malaria cases as compared to the previous malaria season (Fig. 2).

This study investigated the reporting performance of MalariaConnect against the paper-based reporting system during a malaria outbreak in April/May 2017 in Bushbuckridge, Mpumalanga. The primary goal of this study was to retrospectively evaluate the performance and applicability of the paper-based and MalariaConnect reporting tools to deliver appropriate malaria case notifications to the malaria programme during the malaria outbreak. By understanding the capabilities of each system in effectively tracking case numbers and monitoring against epidemic thresholds, the malaria programme can evaluate the benefit of integrating data from the MalariaConnect tool into its Epidemic Preparedness and Response plans.

## Methods

The study period, from 1 January to 18 June 2017, corresponded with an increase in cases and case trend which peaked toward the end of April and beginning of May when the outbreak was declared. Data were extracted from the MIS and MalariaConnect databases on 13 October 2017, to allow time for all paper-based records to be completely recorded and included. Case data collected via paper notifications and captured into the Malaria Information Systems (MIS) were compared against case data captured electronically at the facility level using MalariaConnect was used to assess:

- the time between diagnosis and notification between the paper based reporting with MalariaConnect system (timeliness);
- the proportion of cases reported through the paper system which were also reported in MalariaConnect (completeness);
- the proportion of records notified by MalariaConnect which are valid entries (precision); and
- when each system could have provided sufficient data to identify the malaria outbreak in Bushbuckridge, Mpumalanga.

The data were evaluated using the following metrics: blood smear date, notification date and the time to notification, i.e. when the data are captured from paper-based forms into the MIS from the time of diagnosis or when the MalariaConnect notification was sent. Time to notification was investigated for all facilities which reported cases using MalariaConnect. These same facilities are also required to complete case notification forms for the MIS which corresponding to MalariaConnect notified cases.

To evaluate the comparative performance between the two systems, various approaches were taken. Data validity was investigated using the number of confirmed cases in the MalariaConnect system and all cases in the paper system. Confirmed cases were manually verified by matching cases reported through MalariaConnect to those reported in the paper system. Reporting trends for each tool were compared by observing the frequency that alert and action thresholds were surpassed. To assess timeliness, all paper-reported cases were assessed and matched with MalariaConnect cases using the diagnosis date, creation of MalariaConnect notification, and capture date of paper-based notification. Data completeness was determined by assessing the number of observed reports in the MalariaConnect system, versus the total number of cases reported in the paper system, under the assumption that the totality of cases are reported in the paper-based system. Data precision was assessed by evaluating the number of erroneous case reports in each system e.g. duplicate and test cases reported in MalariaConnect and cases reported with no matching facility in the paper system.

### Data analysis

Data were extracted from the case-based line-lists for each reporting system in Bushbuckridge, Mpumalanga for the 25-week period. All analyses were conducted in SPSS version 20 (IBM Inc., Chicago, U.S.A.). The Chi squared test was used to evaluate if there was a significant difference between indicators extracted from case notifications by paper and those by MalariaConnect. The MIS is the standard reporting tool used to capture all notifiable malaria cases and was treated as the gold-standard system for malaria notifications.

The Epidemic Preparedness and Response standard action threshold (mean case number per week plus 2 standard deviations), as defined by the WHO (“WHO action threshold”), and used by the National Department of Health to identify potential outbreaks, was calculated from the previous 5 years’ case data available from the MIS. Data from the two reporting systems over the 25-week period in this study were assessed against the action threshold to investigate the reliability and timeliness of each tool to detect the malaria outbreak in Bushbuckridge.

## Results

Out of the 59 public health facilities in Bushbuckridge, Mpumalanga [registered hospitals, clinics, Community Health Centres (CHCs), and mobile clinics], 50 were identified as facilities which currently or historically reported malaria cases. Of these 50, seven facilities have not reported malaria cases in the past 2 years via any notification tools and were therefore excluded from further analysis. During the study period (1 January to 18 June 2017), 40 facilities reported cases using the paper tool. This included three cases which were reported in the MIS database but lacked their identifying facility code. However, all three cases were matched to MalariaConnect notifications and were included in further analyses. For MalariaConnect cases, 39 facilities reported using the tool and facilities that were trained but have not used the tool were excluded from further analyses. Two facilities, which reported 1 and 5 cases through the paper system, did not report cases using MalariaConnect and were, therefore, excluded in the analyses.

**Fig. 3:**
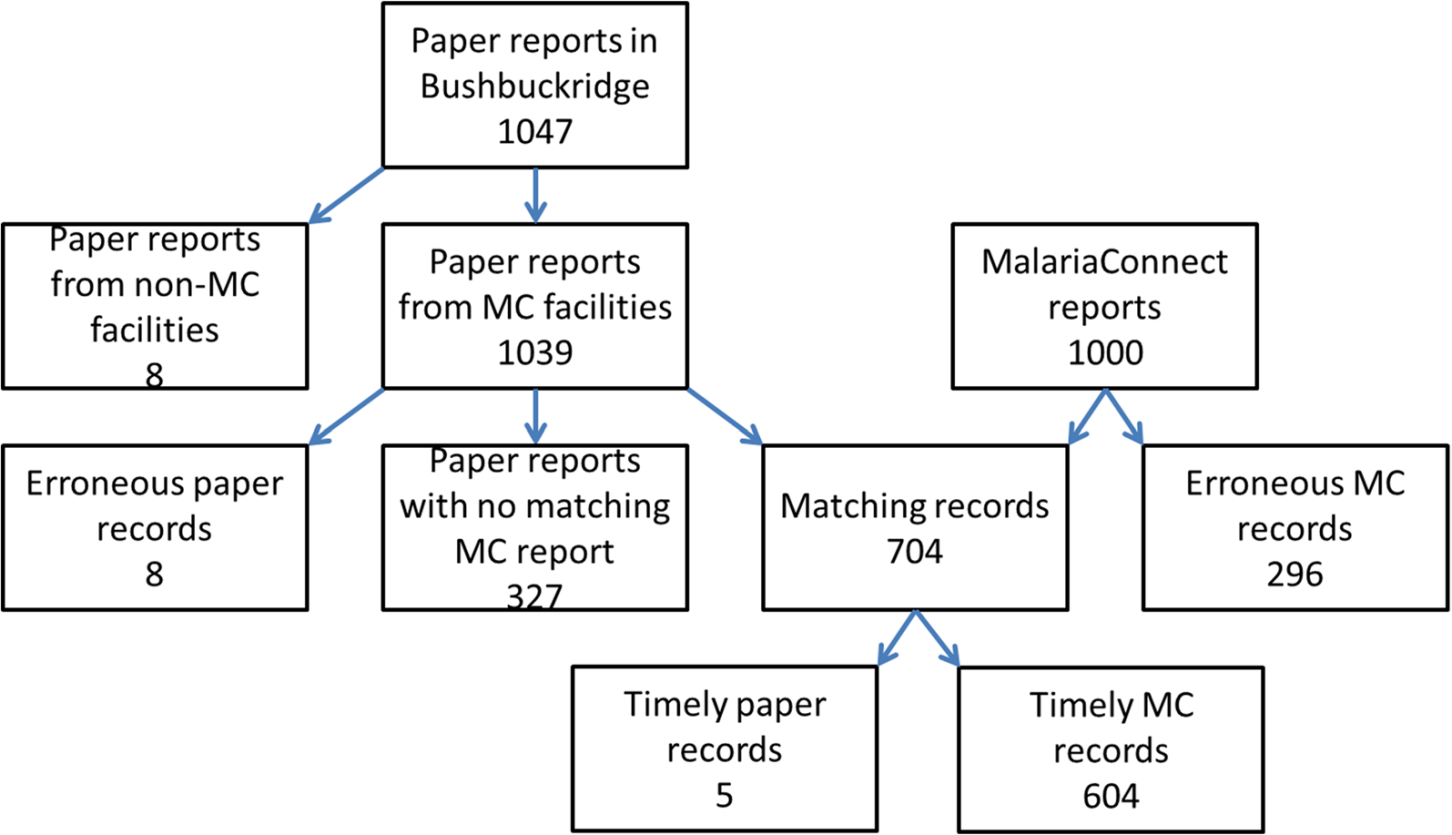
The number of malaria cases reported from the two notification systems during the outbreak period 1 January 2017 to 18 June 2017 in Bushbuckridge, Mpumalanga. MC refers to MalariaConnect and timely reports refers to cases notifications within 24 h

Over the 25-week study period, a total of 1047 malaria cases were notified of which 1039 cases (99.2%) were reported using the paper-based notification system in those facilities that also used MalariaConnect to report cases (Fig. 3). Of the 1039 cases reported by paper, 704 cases (67.8%) were confirmed with MalariaConnect, 330 cases (31.8%) were not reported using MalariaConnect, and 5 cases (0.5%) were identified as duplicate paper reports. There were 1000 notifications received through MalariaConnect during the study period. Of the total MalariaConnect notifications received, 3 cases (0.3%) were tests/suspected training errors, 92 cases (9.2%) were duplicate notifications, 704 cases (70.4%) were confirmed cases and 201 cases (20.1%) were reported through MalariaConnect with no corresponding paper notification.

### Completeness

The MIS case report data was used as the true total number of cases (1039) reported during the outbreak, as the MIS is considered the gold standard, and all malaria cases in the study area should be captured in this system. The completeness of reporting (i.e. confirmed reports found in the database) was significantly higher for the paper system compared to MalariaConnect. The total number of MalariaConnect cases that were matched to cases in the paper system were 704 cases (67.8%) (Table 1).

**Table 1:** Reporting indicators (completeness, precision and timeliness) in the paper-based and MalariaConnect systems

Indicator	Paper-based	MalariaConnect	Definition	
Completeness	–	67.8% (704/1039)	Numerator	Number of confirmed reports found in the database
Notifications from reporting systems match and none are missing			Denominator	Number of cases reported in the paper database
Precision	99.2% (1031/1039)	70.4% (704/1000)	Numerator	Number of erroneous case reports reported
Data free of erroneous records			Denominator	Expected number of cases reported over time
Timeliness	(5/704)	85.8% (604/704)	Numerator	Number of reports received within 1 day
Data are collected and reported in the appropriate timeframe			Denominator	Expected number of reports within 1 day

Completeness of the paper-based reporting system is denoted (–) as this is the benchmark value (100% completeness). All malaria cases are reported through the paper system

**Fig. 4:**
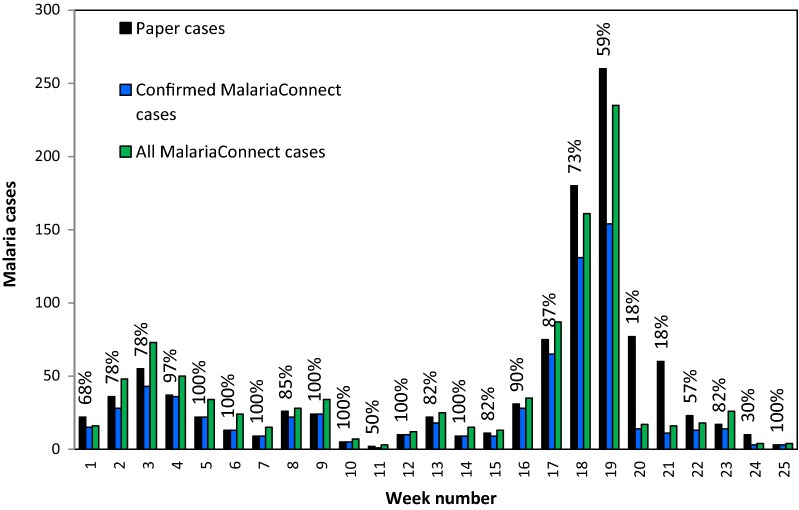
Completeness of reporting of confirmed MalariaConnect case reports (blue, N = 704) with blue percentage indicating the number of confirmed MalariaConnect cases in the paper system (black, N = 1039) per week over the 25-week study period. All MalariaConnect cases reported (including tests, duplicates and neither) are displayed in green (N = 1000). The outbreak was declared in week 19

Figure 4 displays the percentage of confirmed MalariaConnect reports compared to those captured in the paper system, by week, over the duration of the study period. From week 2 to 18, reporting rates with MalariaConnect ranged between 70% and 100%. During week 19 when the outbreak was declared, reporting rates dropped to below 60% and remained low for the following 5 weeks. When including all MalariaConnect case reports, reporting rates increased. Results of the Chi squared analysis indicated that the paper system was more complete than MalariaConnect, as was expected as the paper-based system was assumed to be 100% complete by the starting definitions.

### Precision

Included in the analysis of data precision were the confirmed cases which were matched to the paper system (704 cases) and all test/duplicate and unconfirmed cases reported through MalariaConnect which were not matched to paper case reports (296 cases). Of the 1039 cases reported through the paper system, three cases were from a facility with no name and five cases were duplicates (Table 1). The Chi squared analysis indicated that the paper system was more precise and had less erroneous case reports than the MalariaConnect system and this difference was significant (χ^2^ = 333.9, degrees of freedom, df = 1, p < 0.05) (Table 1).

### Timeliness

**Fig. 5:**
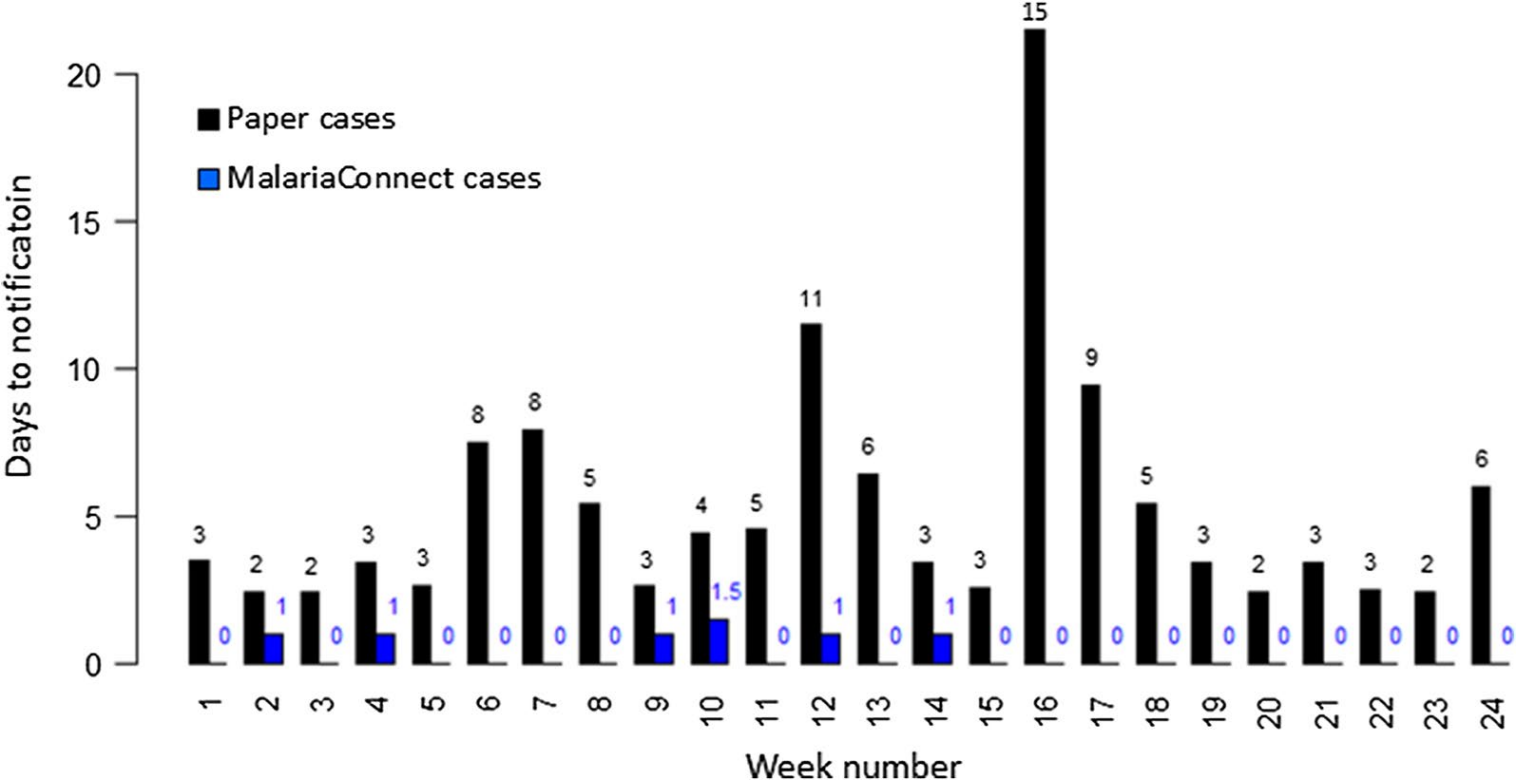
Median number of days between diagnosis and notification for the paper system (black) and MalariaConnect (blue) per week over the 25-week study period. MalariaConnect cases represented here were confirmed in the paper system

A total of 704 confirmed cases were examined to determine timeliness of data reporting in the two systems. Without a confirmed paper report, there is no time stamp on the MalariaConnect case report to determine timeliness. The timeliness of reporting was significantly longer for the paper system compared to MalariaConnect and of the 704 confirmed cases, 5 (0.7%) and 604 (85.8%) cases, respectively, were notified within 24 h of diagnosis. Figure 5 displays the median number of days between diagnosis and notification of the malaria case per week for each reporting system. The mean number of days to report a case through the paper system was 5.65, whereas a case was on average notified through MalariaConnect in 0.63 days. Results of the Chi squared analysis indicated that MalariaConnect performed better in terms of timeliness than the paper system and this difference was significant (χ^2^ = 576.4, df = 1, p < 0.05).

### Data validity

**Fig. 6:**
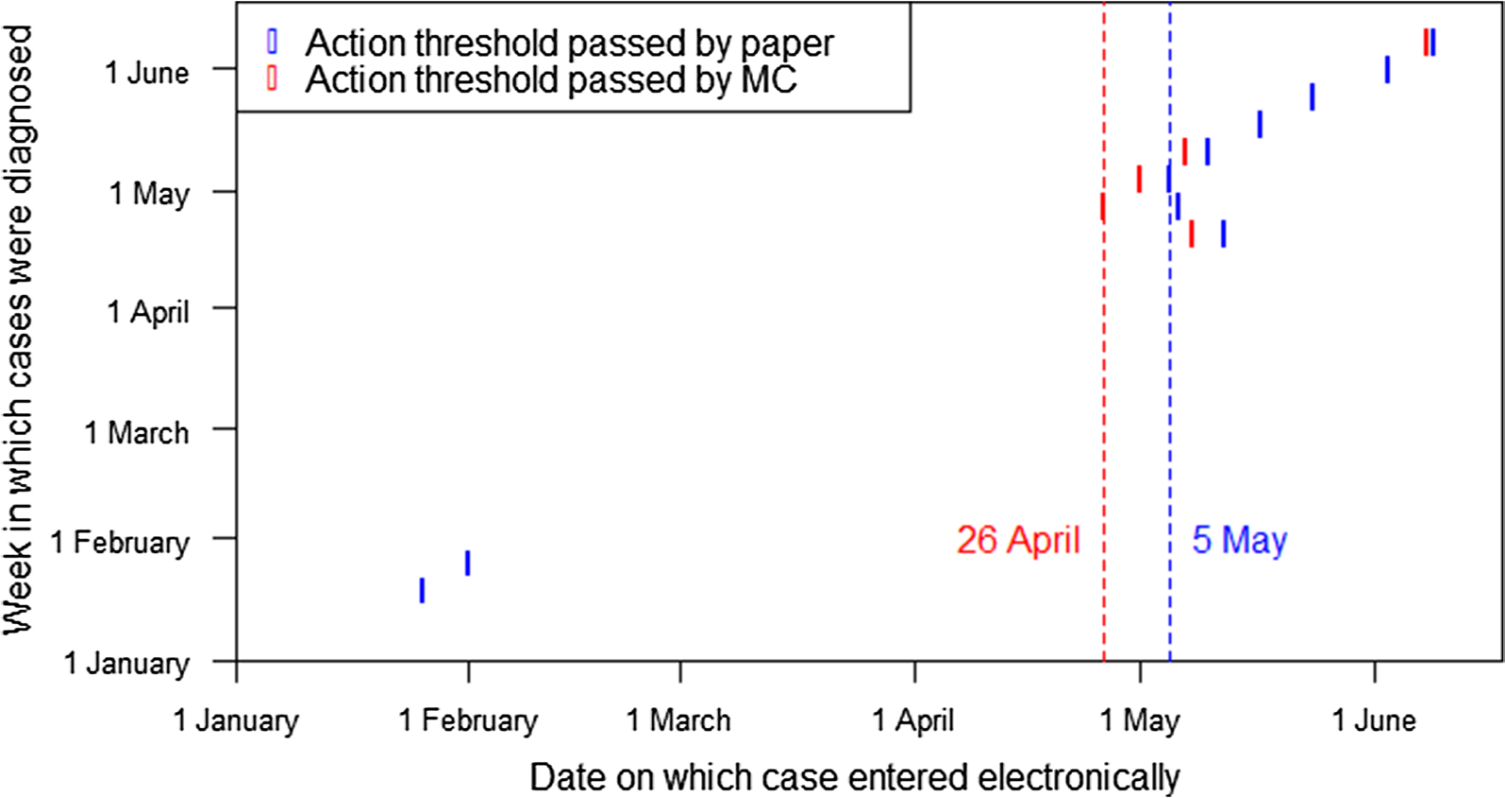
Case notification dates against week in which the case was diagnosed through the paper and MalariaConnect systems (confirmed and all MalariaConnect cases) compared against the World Health Organization and National Department of Health action threshold (mean + 2SD). The week of diagnosis is shown on the vertical axis and each mark on the graph indicates an epidemic week. The position of the mark on the horizontal axis indicates the date on which sufficient cases were notified for it to be identified centrally that threshold had been surpassed. Blue marks indicate when this occurred through the paper system, and red marks when it occurred through MalariaConnect. The red vertical dotted line represents where the MalariaConnect data surpassed the action threshold (26 April, week 17) while the blue line is for the paper system (5 May, week 18)

Validity of the reporting systems, or whether the system provided sufficient data to identify the malaria outbreak, was markedly better using MalariaConnect. Figure 6 demonstrates the link between diagnosis and notification dates. When evaluating case notification dates against WHO action threshold, MalariaConnect was able identify that the action threshold had been surpassed 9 days earlier (26 April vs 5 May) compared to the paper system. The earlier date for surpassing the action threshold within MalariaConnect demonstrates that this system was able to alert users of an oncoming epidemic several days in advance of the paper system. In addition, all MalariaConnect reports consistently reported cases above the action threshold for 12 individual week periods during the study period (weeks 3 to 7, 17 to 21, 23 and 24); using cases reported into the paper system, the action threshold was surpassed for 10 individual week periods (weeks 3 to 5, 18 to 23 and 25).

## Discussion

Reporting on malaria incidence in some countries, including South Africa, has historically been conducted on a weekly basis, allowing for a timelier response to epidemics compared to countries which report on incidence monthly or quarterly [8]. As surveillance systems have been strengthened, the aim of near real-time reporting of cases has come within reach under the objective that surveillance is critical in preventing outbreaks and reducing transmission intensity. Achieving stated national surveillance goals requires prompt and routine reporting of malaria cases in all endemic districts and that all malaria cases should be reported within 24 h of diagnosis.

With the development of information reporting tools such as MalariaConnect which are based on modern communications technology, health facilities can more efficiently and effectively transmit near real-time case data to the relevant malaria programme authorities. Using these widely available mobile reporting tools enables strong disease surveillance by narrowing the communication gap that can impede malaria elimination efforts [9], especially when there is no cost of the service to the user and low cost to the programme [10]. Early detection of epidemics requires this punctual and complete case notification data, which are used to detect the upsurge in malaria cases that signals an oncoming potential epidemic.

In this study, the performance of the MalariaConnect reporting system was evaluated against the paper-based reporting system during a recent malaria outbreak in Bushbuckridge, Mpumalanga, South Africa. All facility staff in Bushbuckridge were trained on the use of MalariaConnect, however some staff did not report through MalariaConnect before or during the outbreak period. This resulted in delays in the initiation of response activities within appropriate time limits, such as case investigation occurring with 48 h and response within 72 h. Overall, however, the MalariaConnect system delivered appropriate malaria case notifications timeously to the malaria programme, tracking case numbers and trends adequately over time, and accurately identifying the increase in malaria cases at the onset of the malaria outbreak. Additionally, the roll-out of the MalariaConnect system has been well received and user acceptability has been reported as 100% among 120 health care workers across health facilities in endemic malaria areas in South Africa [11].

The time between diagnosis and notification of a case is critical to enable malaria programme staff to monitor and rapidly respond to the malaria situation at any given time. During the 2017 outbreak, the slow speed of the paper system failed to promptly detect the increase in case numbers during the outbreak, which resulted in delays in the initiation of epidemic response activities. Furthermore, the paper-based system fell short during the non-outbreak period in 2017 where case reporting was still well beyond the required 24-h period. Coupled with constraints in financial resources, the distances that malaria programme staff must cover in order to transfer paper notifications for data capturing, as well as the act of capturing that paper-based data into the MIS, impede timely reporting and detract from their ability to mount a response.

The importance of rapid detection and response to malaria cases has been repeatedly demonstrated in successful efforts at eliminating malaria in other countries, including Sri Lanka and Turkey. In the case of Sri Lanka, where elimination was declared in 2016, intensive case surveillance activities were implemented, enabling rapid, 24-h reporting of malaria cases by emailing or phoning in on a hotline maintained by the Anti-Malaria Campaign Directorate [12, 13]. In Bushbuckridge, MalariaConnect significantly improved timeliness of reporting of malaria cases during the study period, such that over 80% of confirmed MalariaConnect cases were reported within the 24-h window. Although the official outbreak was declared after cases had exceeded the action threshold, the increase in MalariaConnect notifications alerted individuals at the municipality-level malaria programme in Bushbuckridge to the possibility of an oncoming outbreak, which resulted in increased monitoring of the situation at health facilities.

Over the outbreak period, 201 cases notified through MalariaConnect could not be tied to any corresponding paper report. Because these data were not reported in the paper system, they were not included as the expected number of cases reported during the outbreak when evaluating data completeness. Data precision of paper reports was difficult to ascertain as most of the duplicate paper reports either generated at the facility or captured into the MIS were cleaned and removed prior to the dataset being uploaded into the MIS. Although the completion rate of confirmed MalariaConnect cases was 67.8%, the overall reporting of MalariaConnect, including test and duplicates, was 96%. Despite these reports, the trends in all MalariaConnect cases reported still tracked case trends reported through the paper system. At the peak of the outbreak, MalariaConnect completeness rates declined. Health facility workers may not have sufficient time to report using their mobile phones when case burden is high, workers may have resorted to completing only the paper notification forms as it is the legally required reporting mechanism. An assessment of integrity to determine if there were errors in the paper-based forms and MIS data was beyond the scope of this investigation. Similarly, investigating integrity of MalariaConnect reporting at the facility level was also beyond the scope of this investigation. However, gap assessments in the two surveillance systems should be considered to improve overall completeness, timeliness and precision of data collected.

## Conclusion

The MalariaConnect system provides a near real-time reporting mechanism as compared to the paper reporting system, however better investment is needed at the health facility level to ensure that all cases are notified using MalariaConnect. To improve uptake and use of the tool, a number of activities are recommended, including identification of facilities which underreport or duplicate reporting through the MalariaConnect system, assessment of other barriers to individual use, and the completion of refresher trainings where necessary. As South Africa continues to strengthen its malaria surveillance system, it will be important to identify facilities to ensure they routinely report malaria cases through the correct channels. Regular visits from the malaria programme staff to update health care workers on the value and quality of the data they submit will be helpful in this regard. Dedicated staff in high burden facilities that are trained to report using MalariaConnect may alleviate challenges with reporting through MalariaConnect during periods of high case reporting.

Mobile messaging platforms offer an opportunity to inexpensively and rapidly provide quality surveillance data to national disease programmes. These reporting systems allow for near real-time reporting that can support evidence-based decision making in malaria control and elimination efforts. MalariaConnect provided timely data to detect the increase in cases preceding the outbreak, and although completeness of reporting was not 100%, the mobile tool collected adequate data to declare the outbreak 9 days earlier than the paper system. Strengthening support for and use of mobile reporting tools such as MalariaConnect supports the third pillar of the WHO’s Global Technical Strategy for Malaria, which encourages transforming malaria surveillance into a core intervention [14]. Data made available through the MalariaConnect system should enhance rapid case response activities through data-driven decision making. Documentation of strategies which support surveillance and response activities will benefit malaria endemic countries and contribute to elimination efforts and the prevention of re-introduction.
